# p16(INK4A) expression in invasive laryngeal cancer^☆^

**DOI:** 10.1016/j.pvr.2016.03.001

**Published:** 2016-03-09

**Authors:** Brenda Y. Hernandez, Mobeen Rahman, Charles F. Lynch, Wendy Cozen, Elizabeth R. Unger, Martin Steinau, Trevor Thompson, Maria Sibug Saber, Sean F. Altekruse, Marc T. Goodman, Amy Powers, Christopher Lyu, Mona Saraiya, The HPV Typing of Cancer Workgroup

**Affiliations:** a University of Hawaii Cancer Center, 701 Ilalo Street #239, Honolulu, HI 96813, USA; b University of Hawaii John A. Burns School of Medicine, 651 Ilalo Street #411E, Honolulu, HI 96813, USA; c The University of Iowa, College of Public Health, Department of Epidemiology, 145 North Riverside Drive S447 CPHB, Iowa City, IA 52242-2007, USA; d University of Southern California, Norris Comprehensive Cancer Center and Keck School of Medicine, Departments of Preventive Medicine and Pathology, 1441 Eastlake Avenue MC 9175, Los Angeles, CA 90089-9175, USA; e Centers for Disease Control and Prevention, National Center for Emerging and Zoonotic Infectious Diseases, Division of High-Consequence Pathogens and Pathology, Atlanta, GA 30329-4027, USA; f Centers for Disease Control and Prevention, 4770 Buford Highway NE, Mailstop F-76, Atlanta, GA 30341-3717, USA; g University of Southern California, Norris Comprehensive Cancer Center, 1441 Eastlake Avenue #4420, Los Angeles, CA 90089-9175, USA; h National Cancer Institute, Surveillance Research Program, 9609 Medical Center Drive, Room 4E536, Rockville, MD 20850, USA; i Cedars-Sinai Medical Center, 8700 Beverly Blvd, Room 1S37, Los Angeles, CA 90048, USA; j The Queen׳s Medical Center, Department of Pathology, 1301 Punchbowl St., Iolani 4, Honolulu, HI 96813, USA; k Battelle, 100 Capitola Drive, Suite 200, Durham, NC 27713, USA; l Centers for Disease Control and Prevention, 4770 Buford Highway NE, Atlanta, GA 30341-3717, USA

**Keywords:** Larynx, Laryngeal cancer, Human papillomavirus, HPV, P16(INK4A), P16

## Abstract

We examined p16 expression in tumors from a population-based sample of laryngeal cancer cases diagnosed in the U.S. Samples had been previously genotyped for HPV DNA.

Overall, p16 expression was observed in laryngeal tissue from 8 of 101 (7.9%) cases. p16 expression was observed in 2 of 16 (12.5%) cases previously determined to be HPV DNA positive. The two cases dually positive for p16 and HPV DNA were non-keratinizing SCC and papillary SCC tumors that were positive for genotypes 18 and 35/89, respectively. Positivity for p16 and/or HPV DNA was not associated with 5-year survival (log-rank *p* value=0.55). Our findings support a limited role of HPV in laryngeal carcinogenesis. p16 is not a reliable surrogate for HPV status in laryngeal cancers and is not a predictor of laryngeal cancer survival.

## 1 Introduction

Human papillomavirus (HPV) plays an etiologic and prognostic role in oropharyngeal cancer [1], [2], [3]. Elevated tumor expression of p16(INK4A) (referred to as p16 hereafter), a cyclin-dependent kinase-4 inhibitor, has been well-characterized in oropharyngeal cancer patients and is strongly correlated with HPV positivity. HPV-positivity combined with expression of p16(INK4A) is strong evidence of biologically relevant infection [4].

Unlike oropharyngeal cancers, an etiologic role of HPV in laryngeal and other malignancies of the head and neck has not been definitively established [1], [3]. We recently reported the results of a population-based study to evaluate the genotype-specific prevalence of HPV in invasive laryngeal cancer cases diagnosed in the U.S. [5]. HPV DNA was detected in 31 of 148 (21%) invasive laryngeal cancers; 13 different genotypes were observed. The detection of HPV DNA in tumor tissue, however, is not definitive evidence for causation. The current report examines p16 expression in laryngeal cancer cases in order to further elucidate HPV-related laryngeal cancer development and progression.

## 2 Methods

This study was approved by the CDC Institutional Review Board (IRB) and the IRBs of the University of Hawaii, University of Iowa, and University of Southern California. All patients were diagnosed in 1993–2004 within the catchment area of three population-based cancer registries [5]. Laryngeal cancer cases were selected from patients with pathologically-confirmed tumors. The majority of cases were squamous cell carcinomas of all subsites including the supraglottis, glottis, and subglottis.

De-identified, clinically annotated formalin-fixed paraffin-embedded (FFPE) tissue specimens were obtained from Residual Tissue Repositories (RTR) affiliated with the National Cancer Institute׳s Surveillance, Epidemiology, and End Results (SEER) Program [6], [7]. Through linkage with registry patient and tumor data, tissue specimens were annotated with de-identified demographic, clinical, pathologic, and survival data. Tissue specimens had been previously genotyped for HPV at the CDC laboratories as previously described [5], [8] using the Linear Array HPV Genotyping Test for 37 HPV genotypes (LA, Roche Diagnostics, Indianapolis, IN). The INNO-LiPA HPV Genotyping Assay (LiPA, Innogenetics, Gent, Belgium) was also employed for specimens testing negative for HPV and human beta-globin.

### 2.1 Histologic subtyping by pathologic review

H&E slides of squamous cell carcinoma cases of unspecified subtype, i.e. SCC NOS, were reviewed by a study pathologist (M.R.) for subtype assignment. SCC cases were classified as keratinizing, non-keratinizing, basaloid, verrucous, papillary, and spindle cell.

### 2.2 p16 immunohistochemistry and pathologic review

p16 expression was evaluated via immunohistochemistry. Sections of tumor tissue were obtained from the same FFPE blocks previously used for HPV genotyping. A p16 mouse monoclonal antibody (Santa Cruz Biotechnology, Santa Cruz, CA, USA) (dilution 1:400) was used according to the manufacturer׳s specifications. Slides were read by a study pathologist (M.R.) who was blinded to the HPV status of cases. p16 staining was evaluated based level of staining intensity (mild/weak, moderate, strong), intracellular localization (nuclear, cytoplasmic), staining distribution (patchy, focal, diffuse), and the proportion of tumor cells stained. Specimens exhibiting strong, diffuse nuclear and cytoplasmic staining in ≥70% of tumor cells were considered to be definitively positive for p16 based on established criteria [9], [10].

### 2.3 Statistical analyses

Statistical analyses were conducted using SAS version 9.2. Comparison of p16 expression used the Chi-square statistic. Survival was calculated based on the time period from date of diagnosis to date of death or date of last follow-up. Overall five-year survival by p16 and HPV DNA status was evaluated using the Kaplan–Meier method and the log-rank test. All tests were two-sided and a *p* value <0.05 was considered to be statistically significant.

## 3 Results

The laryngeal cancer study population has been previously detailed [5]. Tumor tissue specimens from 101 of 148 cases from the prior analysis with sufficient tissue for immunohistochemistry were included in the present study. SCC subtypes included 49 (48.5%) keratinizing, 17 (16.8%) non-keratinizing, 9 (8.9%) papillary, 3 (3%) basaloid, 2 (2.0%) spindle cell, and 1 (1.0%) verrucous. A total of 19 (18.8%) of cases remained classified as unspecified SCC and 1 case was a small cell carcinoma.

Eight of the 101 (7.9%) of laryngeal tumors were considered to be positive for p16 based on the criteria of strong, diffuse p16 staining of the nucleus and cytoplasm in ≥70% of tumor cells. Thirty-two cases which exhibited strong, diffuse nuclear and cytoplasmic staining in fewer than 70% of tumor cells and were not considered to be p16 positive. Table 1 compares p16 and HPV DNA status by histologic subtype. Basaloid SCC tumors exhibited the largest proportion of p16 positive tumors (2 of 3). All 3 basaloid tumors were HPV DNA negative. HPV positivity was highest in non-keratinizing (4 of 17) and papillary (2 of 9) SCC tumors. Only 2 of the 26 non-keratinizing and papillary SCC cases were positive for p16 expression.

**Table 1:** p16(INK4A) expression and HPV DNA status by histology of invasive laryngeal tumors.

**Histology**	**p16**		**HPV DNA**	
	**No. positive**	**No. negative**	**No. positive**	**No. negative**
SCC keratinizing (*n*=49)	3	46	8	41
SCC non-keratinizing (*n*=17)	1	16	4	13
SCC papillary (*n*=9)	1	8	2	7
SCC basaloid (*n*=3)	2	1	0	3
SCC spindle cell (*n*=2)	0	2	0	2
SCC verrucous (*n*=1)	0	1	1	0
SCC NOS (*n*=19)	1	18	1	18
Small cell carcinoma NOS (*n*=1)	0	1	0	1
Total	8	93	16	85

**Fig. 1:**
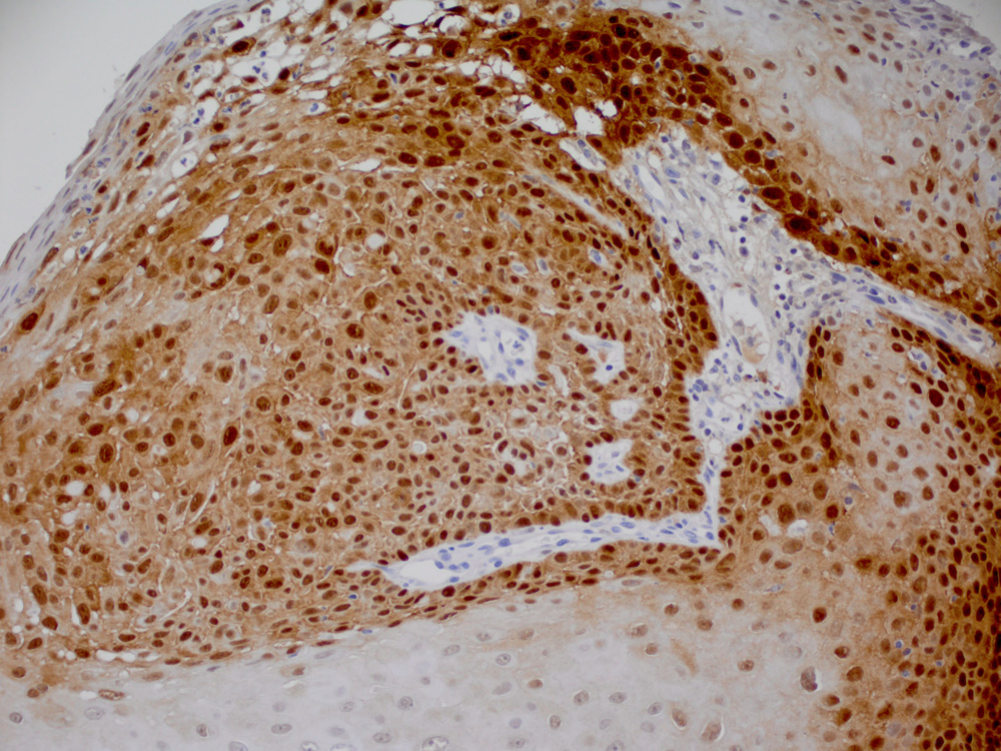
p16 expression in invasive laryngeal (glottal) non-keratinizing SCC tumor positive for HPV 18 DNA. p16 exhibits strong nuclear and cytoplasmic staining of a diffuse pattern in greater than 70% of tumor cells (20×).

**Fig. 2:**
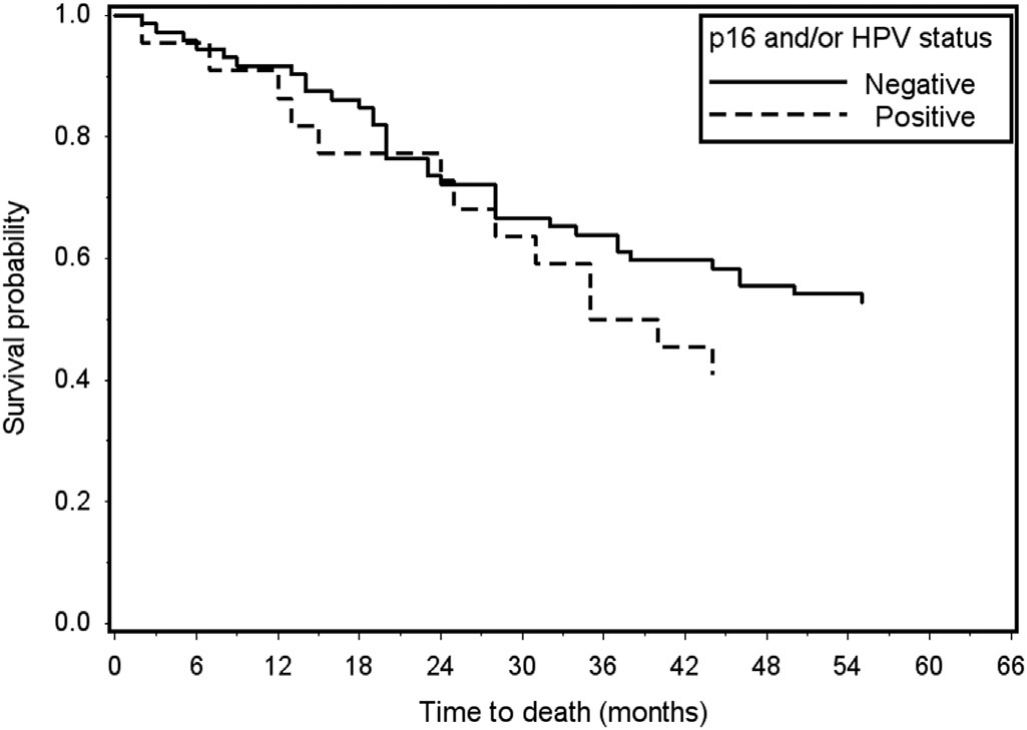
p16 expression & HPV DNA status and overall 5-year survival in invasive laryngeal cancer (*n*=95). There was no difference in overall 5-year survival by positivity for p16 and/or HPV DNA (log-rank *p* value 0.55).

In total, p16 expression was observed in 2 of 16 (12.5%) HPV DNA positive laryngeal cancer cases (Table 2). One p16-positive case was a non-keratinizing SCC positive for HPV 18 (Fig. 1). The second p16 positive case was a papillary SCC positive for both HPV 35 and 89. Both p16/HPV DNA positive cases were localized tumors of the glottis diagnosed in males under age 50. The 14 HPV positive laryngeal cancer cases without p16 expression included glottal and supraglottal tumors of all stages diagnosed in males and females primarily aged 50 and older. Overall survival was evaluated in the 95 cases with vital status and follow-up information. p16 was not associated with 5-year survival when measured based on p16 expression alone (log-rank *p* value=0.84) or positivity for either p16 and/or HPV DNA (log-rank *p* value=0.55) (Fig. 2).

**Table 2:** p16(INK4A) expression in HPV-positive invasive laryngeal tumors.

**p16**	**HPV DNA genotype**	**Subsite**	**SEER stage**	**Histology**	**Grade**	**Gender**	**Age group**
Positive	18	Glottis	Localized	SCC non-keratinizing	2	Male	40–49
Positive	35, 89	Glottis	Localized	SCC papillary	1	Male	40–49
Negative	16	Supraglottis	Regional	SCC non-keratinizing	2	Female	80–89
Negative	35	Glottis	Localized	SCC papillary	1	Female	70–79
Negative	39	Supraglottis	Regional	SCC keratinizing	2	Female	50–59
Negative	16, 54	Supraglottis	Regional	SCC keratinizing	3	Male	70–74
Negative	18, 33	Glottis	Distant	SCC keratinizing	2	Male	65–69
Negative	16	Supraglottis	Localized	SCC keratinizing	2	Male	65–69
Negative	11	Supraglottis	Distant	SCC NOS	3	Female	55–59
Negative	Untyped	Glottis	Localized	SCC verrucous	a	Male	65–69
Negative	16	Supraglottis	Regional	SCC keratinizing	3	Male	70–74
Negative	33	Supraglottis	Regional	SCC keratinizing	2	Female	55–59
Negative	16, 31, 33	Glottis	Localized	SCC keratinizing	2	Male	45–49
Negative	51	Supraglottis	Regional	SCC non-keratinizing	3	Male	75–79
Negative	51	Glottis	Localized	SCC keratinizing	2	Male	60–64
Negative	6	Glottis	Regional	SCC non-keratinizing	2	Female	50–54

a Tumor grade could not be ascertained based on the registry data and secondary pathologic review.

## 4 Conclusions

Our findings support a limited role of HPV in laryngeal carcinogenesis. Fewer than 10% of all laryngeal tumors expressed p16 and p16 expression did not strongly correlate with HPV DNA status. In total, only a fraction (2%) of laryngeal cancers were positive for both p16 and HPV DNA. We previously observed HPV DNA in over 1 in 5 invasive laryngeal cancers. However, detection of HPV DNA alone is not indicative of a clinically relevant infection. In HPV-induced carcinogenesis, the E7 oncoprotein binds and inactivates the retinoblastoma tumor suppressor gene product, pRb [11]. As pRb is a negative regulator of p16, its inactivation results in overexpression of p16 [11]. Therefore, HPV-positivity combined with p16 expression is strong evidence of biologically relevant infection [4]. Our findings of limited correlation of p16 with HPV DNA status contrasts with the few studies that have examined both HPV and p16 in laryngeal cancers. In a study of patients from a single U.S. institution, 65% of p16 positive cases were also positive for HPV DNA [12]. In a pooled analysis of data from two studies, p16 expression was found in 86% of HPV-positive laryngeal cancers [13]. The predominant pattern of p16 expression of laryngeal cancers that we observed was diffuse, strong expression in both the nucleus and cytoplasm. However, for the majority of these cases, fewer than 70% of tumor cells were positive. Wide variation in p16 staining patterns has been observed in head and neck cancers [14]. In general, strong, diffuse nuclear and cytoplasmic staining in the majority (i.e., ≥70%) of tumor cells is considered to be definitively positive for p16 [9], [10]. This p16 expression pattern is seen in the majority of oropharyngeal cancers for which p16 is highly correlated with HPV DNA detection [9], [10], [15]. p16 positivity in the absence of HPV DNA—as observed in a subset of our cases—is consistent with the suggestion that p16 upregulation in laryngeal carcinogenesis may reflect somatic chromosomal alterations unrelated to HPV [16], [17], [18].

Although the sample size was limited, we observed some histologic differences by p16 and HPV status. p16 positivity was most frequently found in basaloid SCC tumors, while HPV DNA positivity was highest in non-keratinizing and papillary SCC tumors. Interestingly, non-keratinizing SCC and, to a lesser extent, basaloid and papillary SCC tumors, are the histologic variants that are most common in HPV-associated oral and oropharyngeal squamous cell carcinomas [19].

The etiologic and prognostic role of HPV in oropharyngeal cancers is well-established [1], [2], [3]. HPV tumor positivity favorably influences outcome, including overall survival, disease-free survival, and recurrence [20], [21], [22], [23], [24], [25], [26], [27], [28], [29], [30], [31], [32], [33], [34], [35], [36]. Unlike oropharyngeal cancers, our findings do not support a prognostic role of HPV in laryngeal cancer. This was consistent for p16 alone and in combination with HPV DNA. We previously observed no survival advantage in HPV DNA-positive laryngeal cancers [5]. Our findings are in agreement with single institution studies that did not observe statistically significant associations of p16 overexpression with laryngeal cancer survival [12], [37], [38].

Our findings support the evidence to date which collectively suggests that, in contrast with the well-established etiologic and prognostic role of HPV in oropharyngeal malignancies, its role in laryngeal cancers is comparatively limited [17]. A major limitation of our study is the lack of information on tobacco and alcohol use, which are the predominant risk factors for laryngeal cancers. Presumably, the majority of laryngeal cancers in the present study were linked to these exposures. Our study findings indicate that any etiologic role of HPV is limited to only a fraction of laryngeal cancers.
